# High-Temperature Oxidation Properties and Microstructural Evolution of Nanostructure Fe-Cr-Al ODS Alloys

**DOI:** 10.3390/ma14030526

**Published:** 2021-01-22

**Authors:** Zhengyuan Li, Lijia Chen, Haoyu Zhang, Siyu Liu

**Affiliations:** School of Materials Science and Engineering, Shenyang University of Technology, Shenyang 110870, China; zhengyli@sut.edu.cn (Z.L.); zhanghaoyu@sut.edu.cn (H.Z.); lsiyuu@outlook.com (S.L.)

**Keywords:** ODS alloys, spark plasma sintering, high-temperature oxidation, microstructure, diffusion

## Abstract

The oxidation behavior and microstructural evolution of the nanostructure of Fe-Cr-Al oxide dispersion strengthened (ODS) alloys prepared by spark plasma sintering were investigated by high-temperature oxidation experiments in air at 1200 °C for 100 h. The formation of Al_2_O_3_ scale was observed by X-ray diffraction (XRD) and scanning electron microscopy (SEM) with energy-dispersive X-ray spectroscopy (EDS) line scans. The oxidation rate of Fe-Cr-Al ODS alloys is lower than that of conventional Fe-Cr-Al alloys, and the oxide layer formed on the Fe-Cr-Al alloy appeared loose and cracked, whereas the oxide layer formed on the Fe-Cr-Al ODS alloys was adherent and flat. This is due to the high density of dispersed nano-oxides hindering the diffusion of Al element and the formation of vacancies caused by them. In addition, the nano-oxides could also adhere to the oxide layer. Besides, the microstructure of the Fe-Cr-Al ODS alloy had excellent stability during high-temperature oxidation.

## 1. Introduction

Fe-Cr-Al alloys are considered one of the Accident Tolerant Fuel (ATF) technologies for future advanced fission [1,2,3]. This material has excellent resistance to high-temperature oxidation due to the formation of alumina film on the surface. Various research has reported that Fe-Cr-Al alloys have a lower oxidation rate and heat-release rate in high-temperature dry air and steam environments than conventional Zr alloys [4,5]. Therefore, the application of Fe-Cr-Al alloys can improve the safety margin of reactor operation.

The high-temperature oxidation resistance of Fe-Cr-Al alloys is significantly affected by the content of Cr and Al elements. The Cr and Al content can be balanced between corrosion resistance, mechanical properties and radiation embrittlement. Usually, the Cr content ranges from 9 to 16 wt.%, and the Al content is around 4 wt.% [6,7,8,9]. In order to increase the high-temperature strength, an oxide dispersion strengthening (ODS) phase (Y_2_O_3_) is added to Fe-Cr-Al alloys [10,11]. However, only adding Y_2_O_3_ alone will lead to a limited increase in the strength as a result of the formation of coarse Y-Al-O particles. To conquer this issue, Zr element is used to form finer Y-Zr-O particles due to the higher binding energies of Y-Zr-O than those of Y-Al-O. Thus, the combined addition of Y_2_O_3_ and Zr elements can significantly improve the high-temperature strength of the Fe-Cr-Al alloy [12,13,14].

For the Fe-Cr-Al base ODS alloy, used as ATF cladding material, the high-temperature oxidation resistance mainly comes from the formation of Al_2_O_3_, which is uniquely influenced by the nanodispersion and the addition of reactive elements such as Y, Ti and Zr [4,15,16]. Merceron et al. [17] compared the oxidation behavior of the Fe-Cr-Al–ODS alloy (MA 956) and Fe-Cr-Al alloy. It is noted that both alloys exhibited excellent oxidation stability at 1200 °C in air. Lipkina et al. [18] measured the mass change over time during high-temperature oxidation by Thermo-Gravimetric Analysis (TGA) in air and steam. As Lipkina et al. suggested, the parabolic oxidation rates in air and steam are almost the same. Maeda et al. [19] studied the influence of Zr on the oxidation of Fe-Cr-Al ODS in air and steam between 1200 and 1500 °C. The results show that proper Zr and O ratios decelerate the oxidation rate. In addition, Unocic [16], Lipkina [18] and Maeda [19] suggested that there is little difference in the oxidation behavior of Fe-Cr-Al ODS alloys in air and water moist environments.

It is well known that many factors can significantly affect the oxidation behavior of the alloy, such as changes in the grain size, precipitation distribution and dislocation density, and atomic diffusion [17]. Regarding the Fe-Cr-Al ODS alloy, in addition to the alloy composition, the fine nano-oxides also play a significant role in the high-temperature oxidation behavior of the Fe-Cr-Al ODS alloy. During the high-temperature oxidation process, the special microstructure of the ODS alloy also evolves. However, there are limited reports focusing on the effect of ODS alloy microstructural evolution on high-temperature oxidation behavior. This work studied the high-temperature oxidation kinetics of the Fe-Cr-Al ODS alloy with Zr addition in air. The aim was to investigate the influence of the Fe-Cr-Al ODS alloy microstructure’s (especially the fine nano-oxides) evolution on the oxidation behavior.

## 2. Materials and Methods

High-purity Fe, Cr, Al, Ti, Zr and Y_2_O_3_ powders were milled in a nominal composition of Fe-15Cr-4.5Al-0.3Ti-0.3Zr-0.3Y_2_O_3_ (wt.%). The Y_2_O_3_ content was at two levels: 0 and 0.3 wt.%. Ball milling was carried out in a Fritsch Pulverisette 5 high-energy planetary mill (Fritsch, Idar-Oberstein, Germany) at 260 rpm for 50 h with a ball-to-powder mass ratio of 10:1 in an argon atmosphere. Spark plasma sintering (SPS) technology (SPS-3.20MK-V, Sumitomo, Tokyo, Japan) was used for subsequent heat curing. The sintering parameters were a 100 °C heating rate to 950 °C for 10 min under a pressure of 40 MPa in a vacuum with a pulsed direct current consisting of a 12 ms power pulse and a 2 ms intermission between pulses. The consolidated specimens were 30 mm in diameter and ~7 mm in thickness. The measured compositions of the consolidated alloys are given in Table 1. The density of the sample after SPS sintering was 97.4% ± 0.2%, and research on the microstructure and properties of ODS alloys with similar compositions prepared by SPS is shown in preliminary reports [20,21,22].

The specimens for high-temperature oxidation were cut into 10 × 5 × 3 mm^3^ pieces by wire electrical discharge machining, and then were ground with grit size 400, 800, 1000, 2000 and 3000 SiC paper and finished with 1 μm diamond polishing paste to obtain a mirror surface. The specimens were then placed in an alumina crucible and subjected to high-temperature oxidation tests in a furnace at 1200 °C for 100 h (included 100 h) with a heating rate of 10 °C/min. In the high-temperature oxidation experiment, only one sample was tested. The samples were taken out of the furnace at different times, weighed when they had cooled to room temperature in air, and then placed in the furnace.

The specimens after high-temperature oxidation were investigated by X’Pert Pro X-ray diffraction (XRD, PANalytical B.V., Almelo, The Netherlands) with a scanning speed of 4°/min and a Cu Kα target. The composition of the oxide layer was determined with a JEOL 6500F scanning electron microscope (SEM, JEOL, Tokyo, Japan) with Energy Dispersive X-ray Spectroscopy (EDS). Electron backscattered diffraction (EBSD) was used to observe the changes in grain size. The EBSD samples were prepared by mechanical polishing and, subsequently, electro-chemical etching in a solution of 20% HClO_4_ + 80% CH_3_CH_2_OH under 20 V at room temperature for about one minute. A JEOL 2100 F transmission electron microscope (TEM, JEOL, Tokyo, Japan) was employed to analyze the evolution of nano-oxides. The TEM specimens were mechanically polished and etched using 4% nitric acid–ethanol. The etched surfaces were coated with a carbon film with a K950X coater (Quorum, Hertfordshire, England), and then chemically etched again in a similar acid solution, and the floating carbon films were retrieved on Ø 3 mm copper meshes.

## 3. Results

### 3.1. High-Temperature Oxidation

**Figure 1:**
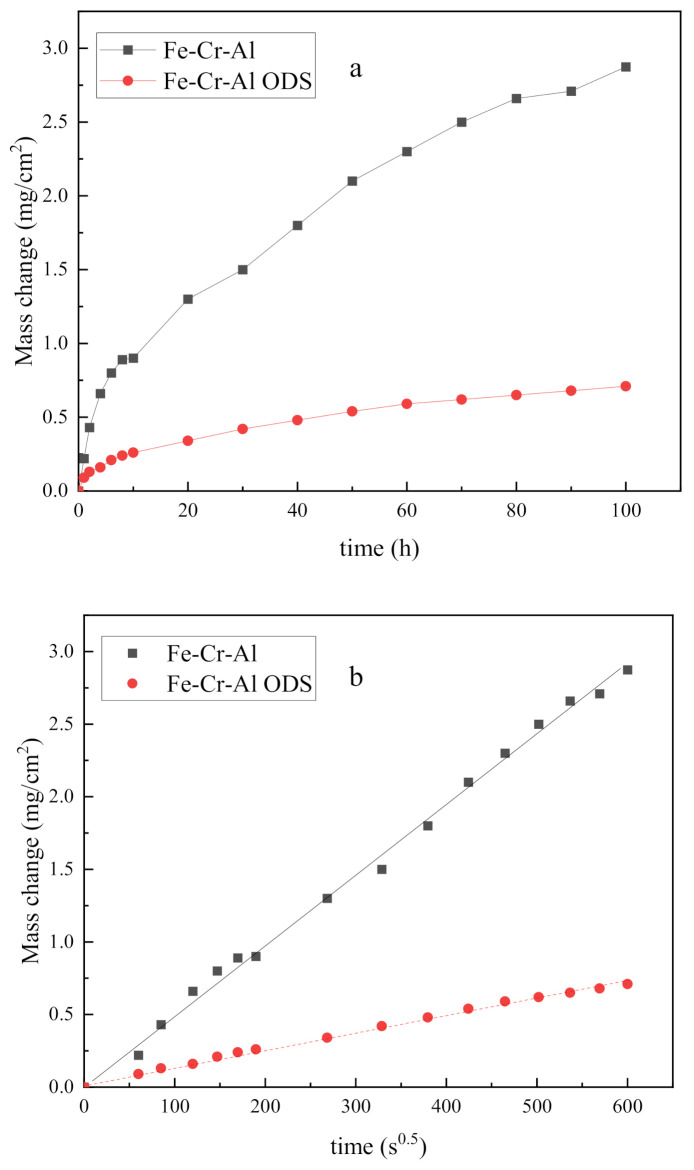
(**a**) Mass change curves and (**b**) the linearity between mg/cm^2^ and s^0.5^ of studied alloys in air at 1200 °C.

Figure 1 shows the mass gain measurements of the Fe-Cr-Al alloy and Fe-Cr-Al ODS alloy oxidized in air at 1200 °C. Although only one sample was measured, reducing the reliability, it still showed obvious regularity, which indicates that the Fe-Cr-Al ODS specimen had better oxidation resistance, which suggests that Y_2_O_3_ addition decelerates oxidation under this condition.

**Figure 2:**
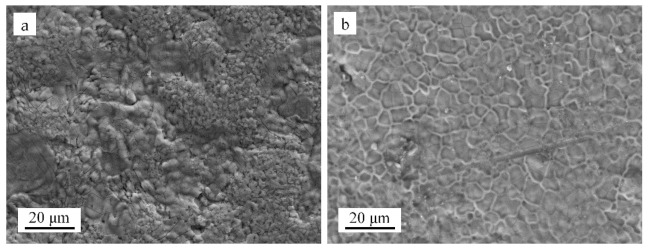
Surface SEM photographs of two alloys oxidized in air at 1200 °C for 100 h: (**a**) Fe-Cr-Al alloy and (**b**) Fe-Cr-Al–ODS alloy.

The surface oxidation morphologies of the Fe-Cr-Al alloy and Fe-Cr-Al ODS alloy are shown in Figure 2. The oxide film of the Fe-Cr-Al alloy was loose and uneven, and there were a lot of pores on the oxide film. However, a compact and smooth oxide film was formed on the surface of the Fe-Cr-Al ODS alloy.

**Figure 3:**
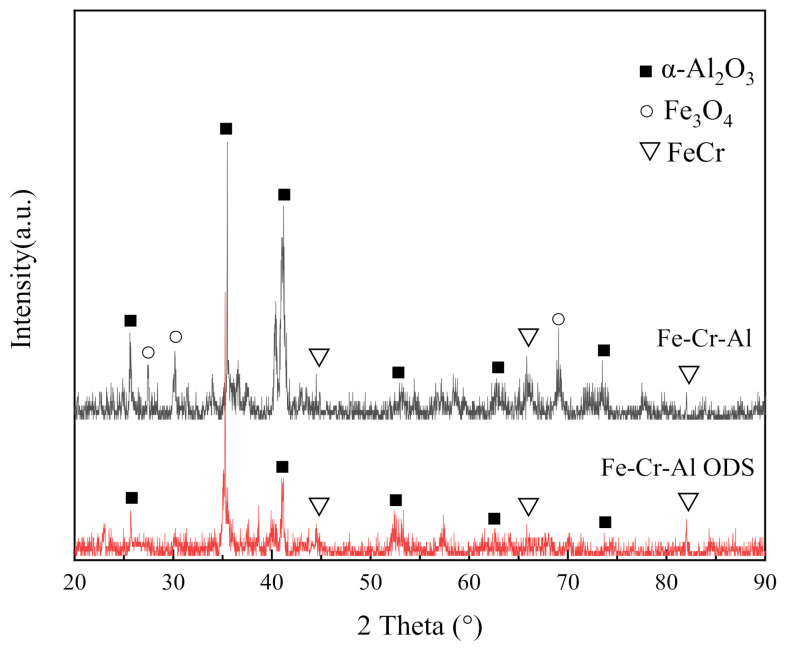
Surface XRD pattern of two alloys after high-temperature oxidation.

Figure 3 shows the surface X-ray diffraction patterns of the Fe-Cr-Al alloy and Fe-Cr-Al ODS alloy after high-temperature oxidation. The relative intensity of the α-Al_2_O_3_ diffraction peaks in the XRD of the two alloys is the highest, which indicates that the main components of the oxide layer after high-temperature oxidation were Al_2_O_3_. For the Fe-Cr-Al alloy, there was a small amount of Fe_2_O_3_ in the oxide layer. In addition, since XRD penetrates the oxide layer to the matrix, the peak of FeCr is displayed for the two alloys.

**Figure 4:**
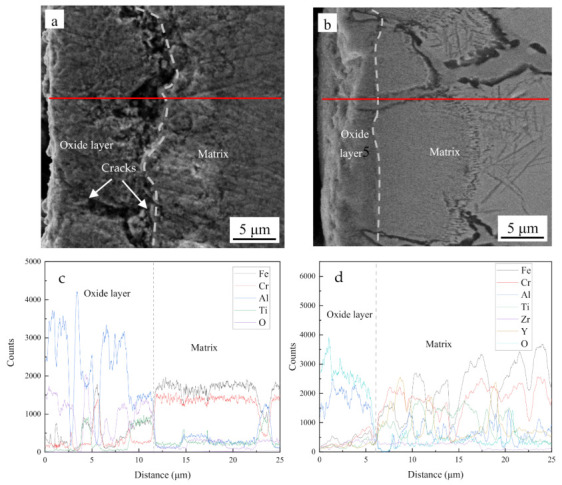
SEM and EDS line of Fe-Cr-Al specimen (**a**,**c**), and Fe-Cr-Al ODS specimen (**b**,**d**).

Figure 4a,b show cross-sections of the two alloys after oxidation treatments at 1200 °C for 100 h. The thickness of the oxide layer of the Fe-Cr-Al specimen was obviously thicker than that of the ODS specimen. The oxide layer thicknesses of the two alloys were about 13 and 6 μm, respectively. Cracks (marked by white arrows) were observed in the oxide layer of the Fe-Cr-Al specimen as shown in Figure 4a, which indicates that O can pass through these cracks resulting in accelerated oxidation t. Upon the addition of Y_2_O_3_ and Zr in the Fe-Cr-Al alloy, the oxide layer became thinner as shown in Figure 4b. The EDS-line scans shown in Figure 4c,d show that the main elements in the oxide layer were Al and O. This suggests the presence of alumina scale on each alloy. In addition, for the Fe-Cr-Al specimen, the matrix near the oxide layer had a lower Al content. 

### 3.2. Microstructural Evolution

**Figure 5:**
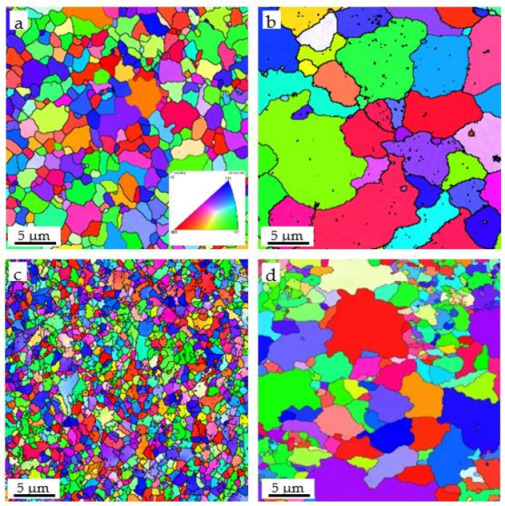
Electron backscattered diffraction (EBSD) analyses of the Fe-Cr-Al specimen before (**a**) and after (**b**) high-temperature oxidation, and Fe-Cr-Al ODS specimen before (**c**) and after (**d**) high-temperature oxidation.

Figure 5 shows the EBSD patterns of the grains of the Fe-Cr-Al alloy and Fe-Cr-Al ODS alloy and their high-temperature oxidation samples. As shown in Figure 5a,c, the two alloys prepared by SPS displayed fine and uniform grains. After high-temperature oxidation, the grains of the two alloys were coarsened. However, whether before or after high-temperature oxidation, the grain size of the Fe-Cr-Al ODS alloy (from 0.33 to 4.42 μm) was smaller than that of the Fe-Cr-Al alloy (from 1.03 to 10.16 μm) due to the nano-oxides.

**Figure 6:**
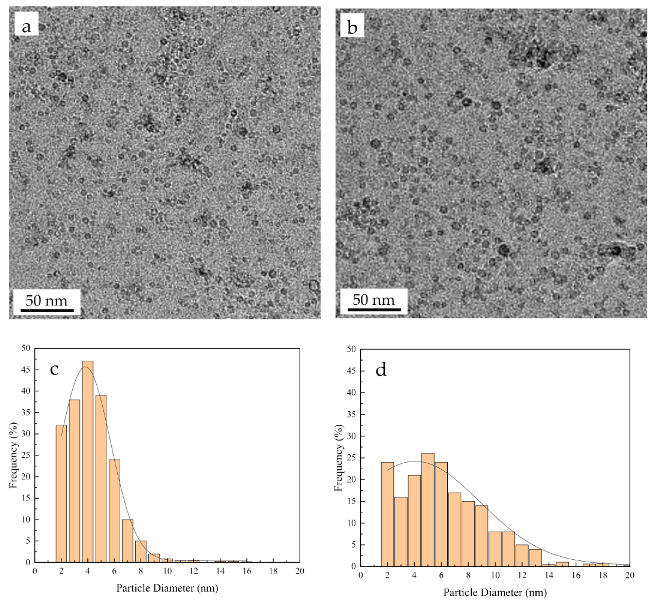
TEM views of nano-oxide particles and their size distributions in Fe-Cr-Al ODS alloys: before (**a**,**c**) high-temperature and after(**b**,**d**) high-temperature oxidation.

Figure 6 shows the TEM images of the nano-oxides near the surface in the Fe-Cr-Al ODS alloy matrix before and after high-temperature oxidation. As can be seen from the Figure 6 that the coarsening of these nanoparticles is limited after long exposure times at 1200 °C, which indicates that the nanoparticles have excellent thermal stability. As Figure 6c,d show, the average sizes of the oxides in the Fe-Cr-Al ODS specimens before and after high-temperature oxidation were about 6.7 and 11.3 nm, respectively. 

## 4. Discussion

**Figure 7:**
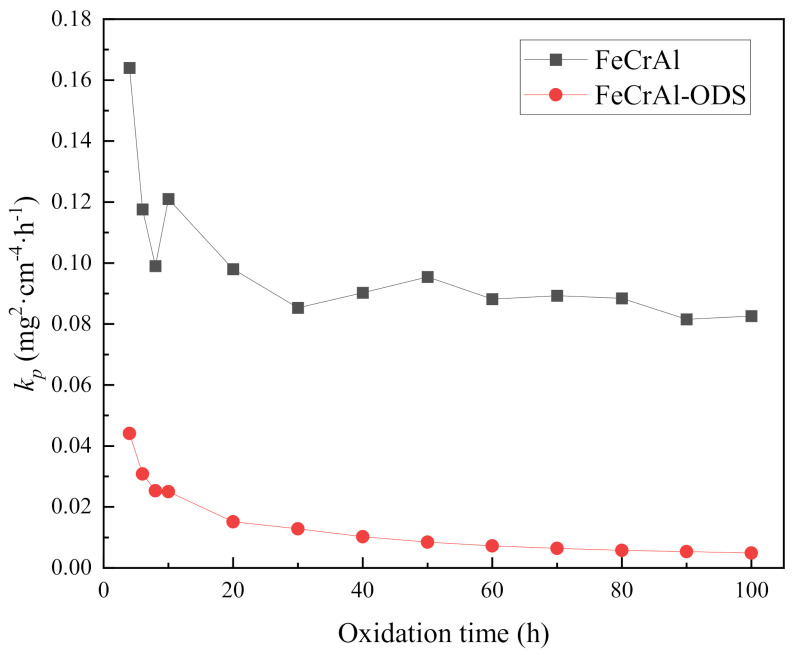
Parabolic oxidation rate *k_p_* with time curves.

When the oxidation reaches steady state, the reaction behavior follows the parabolic rule, and the growth rate of the oxide layer is given by [23]:

*k_p_* = *x*^2^/*t* (1)

where *k_p_* is the parabolic oxidation rate, *x* is the mass gain for each specimen in air and *t* is the oxidation time. The parabolic oxidation rate *k_p_* with time curves are shown in Figure 7. The parabolic oxidation rate was extremely high at the initial stage of the high-temperature oxidation, indicating that the oxidation reaction was very intense. As the oxide film became thicker, it protected the matrix, and the oxidation rate decreased. After 30 h of high-temperature oxidation, the oxidation rate tended to stabilize. However, the parabolic oxidation rate of the Fe-Cr-Al specimen was much higher than that of the Fe-Cr-Al ODS specimen.

The thickness of the oxide scale increases faster in the early stage of oxidation due to the faster oxidation rate. The mass change during initial high-temperature oxidation is expressed as [24]:

2Al(s) + 3/2O_2_(g)—Al_2_O_3_(s) (2)

2Cr(s) + 3/2O_2_(g)—Cr_2_O_3_(s) (3)

3Fe(s) + 2O_2_(g)—Fe_3_O_4_(s) (4)

As the oxidation progresses, the reaction can be expressed as:

2Al + 3/4Fe_3_O_4_—Al_2_O_3_ + 9/4Fe (5)

2Al + Cr_2_O_3_—Al_2_O_3_ + 2Cr (6)

As Equation (2) to Equation (6) show, the growth of the oxide scale requires the continuous diffusion of Al and O. It is generally believed that the elements are more likely to diffuse in defects (vacancies and cracks) and grain boundaries [25,26,27]. As Figure 2 and Figure 4 show, the oxide scale of the Fe-Cr-Al specimen was more dense and thicker, and there were more cracks and porosity than in the Fe-Cr-Al ODS specimen. This is because the rapid oxidation reaction made the oxide scale looser and produced a lot of holes. The metal oxide scale usually has a different expansion coefficient from the alloy matrix. When the temperature changes, the oxide film cracks under the action of thermal tension [28]. This makes it easier for O_2_ to diffuse into the matrix. This resulted in a peak in the oxidation rate curve of the Fe-Cr-Al alloy after 8 h. In addition, the rapid oxidation reaction consumed a large amount of Al element, resulting in the formation of areas with low Al content (as shown in Figure 4c) and a lot of vacancies in the alloy near the oxide scale [29]. As the oxidation reaction progresses, the difference in plastic deformation and the accumulation of vacancies lead to the creation of a fold [26,28].

For Fe-Cr-Al ODS alloys, the grain size is smaller and there are more grain boundaries, as Figure 5 shows; however, the high-density and dispersed nano-oxides can hinder the diffusion of Al. This makes the growth rate of the oxide film not too fast at the beginning of the oxidation, which makes the oxide film denser and enhances the force of bonding with the alloy matrix. 

**Figure 8:**
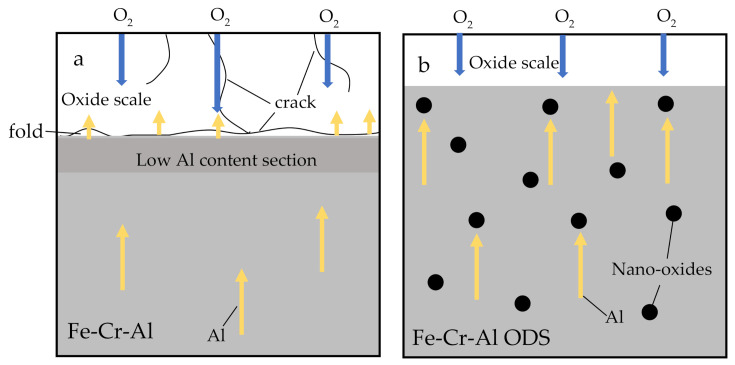
Schematic illustration of mechanism of high-temperature oxidation: (**a**) Fe-Cr-Al alloy and (**b**) Fe-Cr-Al ODS alloy.

In addition, during the oxidation process for 100 h at 1200 °C, the grain sizes of the Fe-Cr-Al alloy and Fe-Cr-Al ODS alloy increased by 9.13 and 4.09 μm, respectively. Additionally, the size of the nano-oxide in the Fe-Cr-Al ODS alloy was coarsened by 68.7% (from 6.7 to 11.3 nm). Table 2 shows the grain size and nano-oxide density, average diameter and volume fraction before and after the oxidation of the Fe-Cr-Al alloy and Fe-Cr-Al ODS alloy. During high-temperature oxidation, high-density nano-oxide hinders the growth of alloy grains due to pinning the grain boundaries [22], and at the same time, it can also prevent the formation of voids by eliminating vacancies at the interface between the internal oxide particles and the matrix [22,30,31]. A schematic illustration of the mechanism of high-temperature oxidation for the two alloys is shown in Figure 8. The black dots in the figure correspond to the Y-rich nano-oxide of the ODS alloy.

## 5. Conclusions

High-temperature oxidation experiments were performed to investigate the oxidation behavior and microstructural evolution of the nanostructure of Fe-Cr-Al ODS alloys prepared by spark plasma sintering. The results obtained are summarized as follows:

1. Although only one sample was measured, the oxidation rate of Fe-Cr-Al ODS alloys is obviously lower than that of Fe-Cr-Al alloys, and the oxide layer formed on the Fe-Cr-Al alloy appeared loose and cracked, whereas the oxide layer formed on the Fe-Cr-Al ODS alloys was adherent and flat.
2. The main components of the oxide layers of the two alloys are Al_2_O_3_. However, due to the existence of voids and cracks in the oxide layer in the Fe-Cr-Al alloys, a small amount of iron oxide was contained in the oxide layer.
3. Due to the pinning effect of nano-oxide, the Fe-Cr-Al ODS alloy exhibited excellent high-temperature stability. This shows that the formation of nano-oxides can not only increase the strength, but also improve the high-temperature oxidation performance.

## Figures and Tables

**Table 1:** The measured chemical compositions of the oxide dispersion strengthening (ODS) alloys (wt.%).

Material	Fe	Cr	Al	Ti	Zr	Y_2_O_3_
Fe-Cr-Al	Bal.	14.6	4.3	0.27	-	-
Fe-Cr-Al ODS	Bal.	14.8	4.2	0.28	0.28	0.31

**Table 2:** Grain size and nano-oxide density, average diameter and volume fraction before and after oxidation of Fe-Cr-Al alloy and Fe-Cr-Al-ODS alloy.

Material	Average Grain Size/μm	Number Density of Particles/m^−3^	Average Particle Diameter/nm	Volume Fraction of Particles/%
Fe-Cr-Al (before oxidation)	1.03	-	-	-
Fe-Cr-Al (after oxidation)	10.16	-	-	-
Fe-Cr-Al ODS (before oxidation)	0.33	3.9 × 10^22^	6.7	0.73
Fe-Cr-Al ODS (after oxidation)	4.42	0.66 × 10^22^	11.3	0.98

## Data Availability

Restrictions apply to the availability of these data. Data was obtained from Northeast University and Shenyang University of Technology and are available from the authors with the permission of Northeast University and Shenyang University of Technology.
